# Effective Connectivity of Right Amygdala Subregions Predicts Symptom Improvement Following 12-Week Pharmacological Therapy in Major Depressive Disorder

**DOI:** 10.3389/fnins.2021.742102

**Published:** 2021-09-13

**Authors:** Yang Xiao, Lei Zhao, Donglin Wang, Shao-Wei Xue, Zhonglin Tan, Zhihui Lan, Changxiao Kuai, Yan Wang, Hanxiaoran Li, Chenyuan Pan, Sufen Fu, Xiwen Hu

**Affiliations:** ^1^Center for Cognition and Brain Disorders, The Affiliated Hospital of Hangzhou Normal University, Hangzhou, China; ^2^Institutes of Psychological Sciences, Hangzhou Normal University, Hangzhou, China; ^3^Zhejiang Key Laboratory for Research in Assessment of Cognitive Impairments, Hangzhou, China; ^4^Affiliated Mental Health Center and Hangzhou Seventh People’s Hospital, Zhejiang University School of Medicine, Hangzhou, China

**Keywords:** amygdala, major depressive disorder, subregion, lateralization, granger causality analysis, treatment

## Abstract

The low rates of treatment response still exist in the pharmacological therapy of major depressive disorder (MDD). Exploring an optimal neurological predictor of symptom improvement caused by pharmacotherapy is urgently needed for improving response to treatment. The amygdala is closely related to the pathological mechanism of MDD and is expected to be a predictor of the treatment. However, previous studies ignored the heterogeneousness and lateralization of amygdala. Therefore, this study mainly aimed to explore whether the right amygdala subregion function at baseline can predict symptom improvement after 12-week pharmacotherapy in MDD patients. We performed granger causality analysis (GCA) to identify abnormal effective connectivity (EC) of right amygdala subregions in MDD and compared the EC strength before and after 12-week pharmacological therapy. The results show that the abnormal EC mainly concentrated on the frontolimbic circuitry and default mode network (DMN). With relief of the clinical symptom, these abnormal ECs also change toward normalization. In addition, the EC strength of right amygdala subregions at baseline showed significant predictive ability for symptom improvement using a regularized least-squares regression predict model. These findings indicated that the EC of right amygdala subregions may be functionally related in symptom improvement of MDD. It may aid us to understand the neurological mechanism of pharmacotherapy and can be used as a promising predictor for symptom improvement in MDD.

## Introduction

Major depressive disorder (MDD) is a widespread and serious mental disease with a range of depressive symptoms and impaired emotional functions as its symbolic features (Hasin et al., 2018; Tottenham et al., 2021). While pharmacological antidepressant therapy is clearly effective, the treatment response is always low and is often hindered by several unsuccessful trials (Saveanu et al., 2015; Kautzky et al., 2021). Considering that treatment prescription based on clinical experience exerts negative effects on occupation, social relationships, and physical health of patients, it is critical that we identify a reliable biological predictor of pharmacotherapy to improve clinical outcome and reduce medical costs (Lam et al., 2016; Fonseka et al., 2018).

Altered functional activities of amygdala were involved in clinical symptoms of MDD, including emotional perception, memory, and reaction, as reported by some meta-analyses (Sergerie et al., 2008; Diener et al., 2012; Miller et al., 2015). As the core of the emotional circuitry in the brain, the amygdala plays a key role in the pathway among emotional feelings and responses (LeDoux, 2000). Various neuroimaging studies have focused on ascertaining the relationship between the functional changes of the amygdala and the changes of clinical symptoms in MDD patients (Arnone, 2019). According to a facial emotion recognition paradigm-based functional magnetic resonance imaging (fMRI) study, MDD patients showed low reactivity in amygdala compared to controls at baseline and increased toward “normalization” after treatment (Ruhé et al., 2012). A resting-state fMRI (R-fMRI) study also reported that antidepressant treatment changed the amygdala abnormal functional connectivity (FC) in adolescents with MDD (Cullen et al., 2016). Notably, these literatures suggested that changes of the amygdala functions may signify neural changes behind pharmacotherapy in MDD, but they ignored the heterogeneousness and lateralization of amygdala (Sah et al., 2003).

Based on cytoarchitectonic characteristics, some researchers (LeDoux, 2000, 2007; Amunts et al., 2005) suggest that the amygdala is composed of three subdivisions: centromedial amygdala (CM), laterobasal amygdala (LB), and superficial amygdala (SF). Recent neurobiological studies have revealed that these three amygdala subregions may have unique connectivity and distinct functional profiles (McGaugh, 2004; Hofmann and Straube, 2019; Michely et al., 2020). The LB subregion is usually viewed as the sensory interface of the amygdala, which is responsible for receiving the sensory input from the thalamus and auditory cortex, mainly including the auditory inputs (LeDoux, 2003). The CM is regarded as the output regions, which play an important role in generating the behavior response for emotion stimulation (Phelps and LeDoux, 2005). It has been found that the SF in the amygdala has connections with the hypothalamus, frontal cortex, and hippocampus and appears to regulate the visceral function related to emotional stimulation (Price, 2003). A study of amygdala subregion function demonstrated that both LB-prefrontal cortex and CM/SF-brainstem connectivity abnormalities exist in MDD (Tang et al., 2019). In another study, MDD exhibited dysfunctional amygdala subregions to frontal cortex circuitry, but no difference was found when using the whole amygdala as seeds (Qiu et al., 2018). These finding indicates that the amygdala has partially separated information processing between amygdala subregions, and it is necessary to divide the amygdala into different subregions. On the other hand, a growing body of studies have emphasized the different roles for the right and left amygdala in emotion processing; that is, there is a lateralized activity pattern of the amygdala (Baas et al., 2004). For instance, some researchers suggested that the right amygdala may be more involved in the analysis of visual information, and it will be activated more strongly when visual stimulation appeared (Markowitsch, 1998). Moreover, the right amygdala is faster, shallower, and more automated than the left amygdala in processing information (Baas et al., 2004). The right amygdala may be the first to participate in emotional analysis and then quickly becomes habituated for negative stimulation (Wright et al., 2001). The main function of habituation is to limit the use of attention resources to stimulations; impaired habitual function will easily lead to depression-related sustained negative emotions and thinking rumination due to a number of negative emotional experiences that cannot be habituated (Wright et al., 2001). Intriguingly, previous studies have revealed more effect on the right amygdala after treatment, hence implying the clinical potential of the right amygdala in therapy (Suslow et al., 2010). To some extent, the aforementioned findings indicate that the analysis of right amygdala subregions is more promising for elucidating the mechanisms of pharmacotherapy.

Effective connectivity (EC) is an effective technique to characterize the brain information flow in the interacting brain regions; furthermore, EC can detect the direction of information and describe the casual influences exerted among different brain regions, which have facilitated the identification of abnormal intrinsic brain activity in various neurological and neuropsychiatric diseases (Deshpande and Hu, 2012). Granger causality analysis (GCA) is a popular method to estimate EC using responses from time-series data in different regions to infer the direction and intensity of the causal influence of regional neural activity (Goebel et al., 2003; Hamilton et al., 2011). Prior studies in MDD treatment have taken advantage of the GCA technique to investigate the effect of electroconvulsive therapy (ECT), and the result indicated that the amygdala subregion EC can be used as a predictor of the treatment effect of ECT (Wang et al., 2017). The information flow of amygdala subregions may underlie the clinical symptom improvement of MDD. Exploring MDD symptom improvement caused by pharmacotherapy in EC of amygdala subregions is clinically meaningful and provides directional information of brain function, which cannot be detected by FC. However, very few studies investigate whether the EC of right amygdala subregions at baseline can predict medication efficacy.

Here, we have three aims: (1) to determine the abnormal EC of right amygdala subregions in MDD patients; (2) to explore the relationship between the variance in EC and symptom improvement before and after 12-week pharmacological treatment using longitudinal analysis; and (3) to predict symptom improvement using the EC strength of right amygdala subregions at baseline. As far as we know, few studies have focused on the subregions of the right amygdala and using the GCA to investigate whether the EC can effectively predict the symptom improvement in MDD patients. Given some previous evidence in the analysis of the amygdala implicating that the function of the amygdala is linked with antidepressant interventions (Fonseka et al., 2018), we thus hypothesized that pharmacotherapy would normalize the abnormalities in EC of right amygdala subregions, and right amygdala subregion-based EC strength at baseline is able to predict the symptom improvement of MDD patients after 12-week pharmacotherapy.

## Materials and Methods

### Participants and Study Design

A total of 70 MDD patients (age: 26.93 ± 9.14 years, 21 males/49 females) and 43 sex- and age-matched healthy controls (HCs) (age: 29.42 ± 12.56 years, 16 males/27 females) were recruited from the Department of Psychiatry of Hangzhou Seventh People’s Hospital and the Department of Psychiatry at The Affiliated Hospital of Hangzhou Normal University. More detailed information of the participants was summarized in Table 1. All patients were interviewed by certified psychiatrists, and 24-item Hamilton Rating Scale for Depression (HAMD) was used to assess the severity of depression symptoms. All patients met the following exclusion criteria: (1) currently pregnant or lactating; (2) serious suicidal tendency; (3) severe medical or neurological illness; (4) material dependence, including tobacco, alcohol, or other psychoactive substances; or (5) metallic implants or other contraindications to MRI. All the research procedures were carried out in accordance with the Helsinki Declaration of Ethical Principles and approved by the local Institutional Review Boards of Hangzhou Normal University. All subjects provided written informed IRB-approved consent before participating.

**TABLE 1:** Demographic data and group differences.

Characteristics	MDD (Mean ± SD)	HC (Mean ± SD)	*t*/χ2 value	*p*-value
**Sex (M/F)**	70 (21/49)	43 (16/27)	0.63	0.43^a^
**Age (years)**	26.93 ± 9.14	29.42 ± 12.56	−1.22	0.23^b^
**HAMD scores**	28.06 ± 6.67 (70)	11.41 ± 7.09	11.76	<0.001^b^
Before pharmacotherapy	27.78 ± 6.70 (36)			
After pharmacotherapy	11.42 ± 7.09 (36)			
**Durations of illness (months)**	7.56 ± 12.74			
**On-medication (n patients)**				
SSRIs	36			

*MDD, major depressive disorder; HC, healthy control; SD, standard deviation; M, male; F, female; HAMD, 24-item Hamilton Rating Scale for Depression; SSRIs, selective serotonin reuptake inhibitors.*

*^*a*^The *p*-value was obtained by a chi-square test.*

*^*b*^The *p*-value was obtained by a two-tailed two-sample *t*-test.*

**FIGURE 1:**
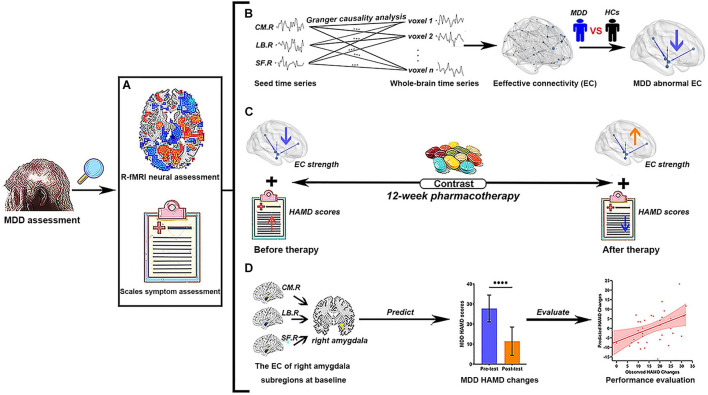
Study flowchart of current research. **(A)** Two different MDD assessment methods were used in the present study. **(B)** The EC analysis of right amygdala subregions using Granger causality analysis. We explore abnormal right amygdala subregion-based EC in MDD patients by the comparison of R-fMRI data between MDD and HCs. **(C)** The contrast of the variance of EC strength and the changes of HAMD scores of MDD patients before and after pharmacotherapy, respectively. **(D)** The voxel-wise EC strength of right amygdala subregions at baseline is taken as feature sets to predict the changes of HAMD scores in MDD patients. The Spearman’s rank correlation between predicted HAMD scores changes and observed HAMD scores changes is used to evaluate the prediction power of current predictors. MDD, major depressive disorder; HC, healthy controls; R-fMRI, resting-state functional magnetic resonance imaging; CM, centromedial amygdala; LB, basolateral amygdala; SF, superficial amygdala; EC, effective connectivity; HAMD, 24-item Hamilton Rating Scale for Depression; L, left hemisphere; R, right hemisphere. Different color arrows indicate the change direction of studied indicators compared with the previous stage: cool colors indicate decrease and warm colors indicate increase. ****p < 0.0001.

The study design flowchart is shown in Figure 1. The assessment of MDD patients included R-fMRI neural and scale symptom assessment in the current study. Before pharmacotherapy, all patients with MDD and HCs completed resting-state fMRI scan and HAMD scale to get the time series of each voxel in the whole brain and HAMD scores; meanwhile, we used GCA to calculate the EC between the time series of three amygdala subregions and whole brain voxels, and to explore the abnormal EC of right amygdala subregions in the MDD group through between-group comparison. After pretests, MDD patients then began to receive antidepressant treatment with typical selective serotonin reuptake inhibitors (SSRIs). The medication doses were prescribed and adjusted by the treating clinicians according to routine clinical practice and followed the recommended dose ranges. It is worth nothing that we chose the same kind of drugs (SSRIs) to reduce the heterogeneity of antidepressant drugs, which are recognized and close to homogeneous interventions in scientific research. After 12 weeks of treatment, the remaining 36 (51.43%) of the 70 MDD patients were invited to return to enter another identical fMRI scan and HAMD scale; the EC strength and HAMD scores of the MDD group were obtained again, and we performed a longitudinal analysis to explore the changes of EC strength and HAMD scores in pre- and post-test of treatment, respectively. Finally, we used the voxel-wise EC strength of between right amygdala subregions at baseline to predict symptom improvement, which was defined as the changes of HAMD scores (HAMD scores in pre-test – HAMD scores in post-test), through a regularized least-squares regression using the Least Absolute Shrinkage and Selection Operator (LASSO) algorithms-based machine learning approach, and Spearman’s rank correlation analysis was used to evaluate the model predictive power. The HC participants did not take any medicine and received only one fMRI scanning.

### Image Acquisition and Preprocessing

Baseline imaging data for 70 MDD patients and 43 HCs were collected before pharmacotherapy to determine neural alterations in depressed individuals, and follow-up images of 36 (51.43%) MDD patients were acquired after 12-week pharmacological therapy. Baseline and follow-up imaging data were obtained by a Siemens MAGNETOM Allegra syngo 3.0T MR Scanner (Siemens AG, Medical Solutions, Erlangen, Germany) at the Center for Cognition and Brain Disorders at Hangzhou Normal University. Functional images were collected by using a T2^∗^-weighted gradient-recalled echo-planar-imaging (EPI) sequence, which has the following parameters: 33 axial slices with a slice thickness = 3 mm, repetition time (TR) = 2,000 ms, echo time (TE) = 30 ms, field of view (FOV) = 220 mm × 220 mm, flip angle = 90°, matrix = 64 × 64, and number of total volumes = 240. A high-resolution T1-weighted structural image in the sagittal orientation is obtained by using magnetization-prepared rapid gradient echo (MPRAGE) sequence. The participants were told to relax with their eyes closed but not fall asleep, and keep motionless during the scanning as much as possible.

The preprocessing of image data was conducted using a combination of the DPABI software^1^ (Yan et al., 2016) and a custom code written in MATLAB (The MathWorks, Inc., Natick, MA, United States). The first 10 functional volumes were discarded to stabilize the scanner signals and ensure that the participants adapt themselves to the circumstances. The remaining 230 images were performed by slice timing correction and then realigned to the first volume for head motion correction. The head motion information is recorded by estimating the translations in each direction and the rotations in angular motion about each axis for each of the consecutive volumes. All participants exhibited a maximum displacement of less than 2.5 mm in the x, y, or z directions and an angular motion of less than 2.5° for each axis. To further control the confounding influence of head motion, the framewise displacement (FD) across time points was calculated for further analysis (Power et al., 2012). The residual effects of Friston-24 motion parameters and signals of white matter and cerebrospinal fluid were controlled by linear regression. The corrected images were normalized into standard Montreal Neurological Institute space (resampling voxel size = 3 mm × 3 mm × 3 mm). The images were smoothed with a 6-mm full-width at half-maximum Gaussian kernel. To reduce the effect of the physiological artifacts, we removed several sources of nuisance signals [six motion parameters, white matter signal, and cerebrospinal fluid (CSF) signal] from the smoothed images through linear regression. After band-pass filtering (0.01–0.1 Hz), the “scrubbing” cut method was employed to remove the “bad” time points using Piecewise Cubic Hermite interpolation, and the threshold is 0.5 mm (Liao et al., 2018).

### Effective Connectivity Analysis of Right Amygdala Subregions

**FIGURE 2:**
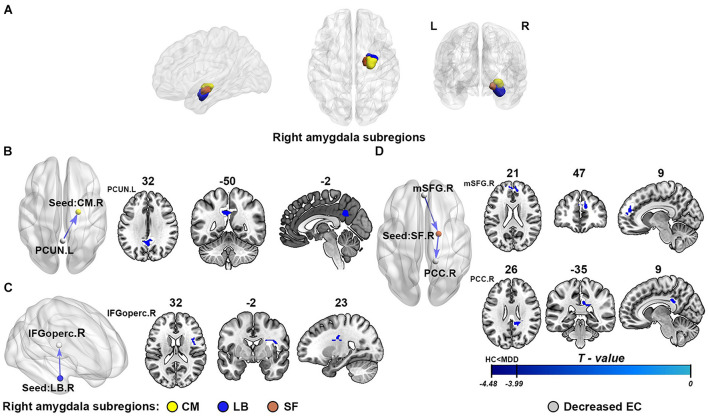
The results of effective connectivity analysis of right amygdala subregions. **(A)** The subregions of the right amygdala: the centromedial amygdala (CM; yellow), laterobasal amygdala (LB; purple), and superficial amygdala (SF; red). **(B–D)** Represent the illustration of the abnormal effective connectivity of MDD in three different right amygdala subregions, respectively: CM, LB, and SF. The cool color arrows indicate significant decreased information flow of target brain regions in MDD. The gray indicates the specific abnormal brain regions, and the yellow/purple/red represents right CM/LB/SF, respectively. EC, effective connectivity; CM, centromedial amygdala; LB, basolateral amygdala; SF, superficial amygdala; PCUN, precuneus; IFGoperc, opercular part of inferior frontal gyrus; mSFG, medial superior frontal gyrus; PCC, posterior cingulate cortex; L, left hemisphere; R, right hemisphere.

Following some existing literatures, we defined three right amygdala subregions by using cytoarchitectonically defined probabilistic maps and select the masks of regions of interest (ROIs), including CM, LB, and SF as provided within the SPM Anatomy toolbox. All ROI masks of amygdala subregions will serve as the seeds for subsequent EC analysis. The detailed locations of the selected ROIs are shown in Figure 2A. The right amygdala subregions were showed using the BrainNet Viewer package (Xia et al., 2013).

In order to acquire the resting-state EC map of each subregion of the right amygdala, the GCA method was used to describe the EC. The GCA is a method based on multiple linear regression, which is used to study whether the current value of time series Y is correctly predicted by a past value of another time series X, and if the combination of the time series X and Y past value could more accurately estimate the time series Y current value than the time series Y past value alone, then time series X has a Granger casual influence on series time Y (Roebroeck et al., 2005). In the present study, voxel-wise GCA was implemented by the DynamicBC toolbox (Liao et al., 2014). The time series of the three right amygdala subregions was defined as the seed time series X, and the time series Y represents the time series of the rest of brain voxels. The residual-based GCA model was carried out to investigate the EC between amygdala subregions and each voxel of the whole brain. Finally, residual-based F was normalized to a Z score using a custom code written in MATLAB for each voxel to improve the normality of F for further statistical analysis (Zang et al., 2012).

### Predictive Model Definition and Evaluation

To explore whether the effective connectivity of right amygdala subregions might serve as useful predictors for symptom improvement in MDD, a regularized least-squares regression using LASSO algorithms combining with a nested cross-validation predicted model was employed. We repeatedly analyzed our current data using two different cross-validation strategies, leave-one-out cross-validation (LOOCV) and 10-fold cross-validation (10-fold CV), for internal validation, and added it to the predicted model to improve robustness and repeatability of our conclusions. The predicted model was carried out by using MATLAB; the dependent variable is the HAMD changes, and the independent variables included the EC between one right amygdala subregion and all voxels in the other two subregions at baseline. The LASSO regularization uses the method based on the L1 constraint to perform the selection of correlated variables and prevent unimportant features from resulting in an overfitting problem (Witten and Tibshirani, 2011), and the cross-validation strategy was adopted to improve the generalization ability of the model. The model was fit to the relationship between the EC strength of right amygdala subregions and HAMD changes in each feature set of n participants (where n is the number of participants, *n* = 36 in this study), which was repeated k times (where k is the number of the fold in cross-validation, including 36 and 10 in the current study). In each cross-validation fold, we set the values of alpha = 1 and use the internal 10-fold CV to select the best LASSO regularization parameter lambda (λ), which controls the number of features selected, and calculated the predicted HAMD changes of the left-out subject. After the cross-validation, the predicted HAMD changes were achieved, and a Spearman’s rank correlation between observed HAMD changes and predicted HAMD changes was used to evaluate the model predictive performance. The non-parametric *p*-value was calculated based on 10,000 permutation tests.

### Statistical Analysis

In baseline MDD patients and HCs, through independent-sample *t*-test and regressed covariates of age, sex, and head motion, the differences of EC strength in right amygdala subregions between MDD and HC groups were examined. To reduce the type I error, the family-wise error (FWE) correction was conducted using the Gaussian random field (GRF) theory, and the significance threshold of FWE correction was set to *p* < 0.001 at the voxel level and FWE correction at the cluster level to *p* < 0.0167 (0.05/3) through Bonferroni correction.

To obtain the symptom change after pharmacotherapy, we utilized paired-sample *t*-test to compare the HAMD scores of MDD patients in before and after treatment, and the threshold for significance was set as *p* < 0.05. The HAMD scores of these patients will be used as an index to evaluate the clinical symptom improvement of MDD. Paired-sample *t*-test was also used to assess the variance of abnormal EC strength in right amygdala subregions found in between-group comparison before and after pharmacotherapy. The statistical threshold was set at *p* = 0.05 and using false discovery rate (FDR) correction for multiple comparisons.

To further determine whether the neural changes are related to symptom improvement and make further analysis meaningful, we performed Pearson’s *r* correlation analysis between the variance of abnormal EC strength and HAMD score changes in the MDD group. The significance level threshold was set at *p* < 0.05.

## Results

### Effective Connectivity Analysis

As shown in Figure 2 and Table 2, compared with the healthy controls, the patients with MDD showed attenuation of EC strength, mainly including three inhibitory pathways: (1) from the left precuneus (PCUN) to the right CM, (2) from the right LB to the right opercular part of the inferior frontal gyrus (IFGoperc), and (3) from the right medial superior frontal gyrus (mSFG) to the right SF to the right posterior cingulate cortex (PCC). There were no significant between-group differences in the causal outflow from the right CM to other brain regions and the causal inflow from other brain regions to the right LB.

**TABLE 2:** Brain regions with significant differences in right amygdala subregions seeded-EC between MDD and HC.

Seed region	Anatomical region	Cluster size (voxels)	Peak *T*-value	MNI (XYZ) coordinates
**Right CM**				
Input regions	PCUN.L	55	−3.99	0, −54, 33
**Right LB**				
Output regions	IFGoperc.R	48	−4.48	39, 0, 21
**Right SF**				
Input regions	mSFG.R	41	−3.99	9, 48, 21
Output regions	PCC.R	48	−4.41	15, −42, 27

*The statistical threshold used the GRF theory [single-tailed, voxel-level *p* < 0.001, cluster-level *p* < 0.0167 (0.05/3, Bonferroni correction)]. MNI, Montreal Neurological Institute; CM, centromedial amygdala; LB, basolateral amygdala; SF, superficial amygdala; PCUN, precuneus; IFGoperc, opercular part of inferior frontal gyrus; mSFG, medial superior frontal gyrus; PCC, posterior cingulate cortex; L, left hemisphere; R, right hemisphere.*

### Longitudinal EC Analyses Following 12-Week Pharmacotherapy

In the MDD group, 36 (51.42%) patients completed both before and after treatment clinical assessment and MRI scanning. The primary clinical symptom improvement was assessed by 24-item HAMD scores before and after 12-week pharmacological treatment. Following antidepressant treatment, the results showed that the symptom of MDD has a significant improvement (*t* = 11.764, *p* < 0.0001, paired-sample *t*-test) after the pharmacotherapy.

**FIGURE 3:**
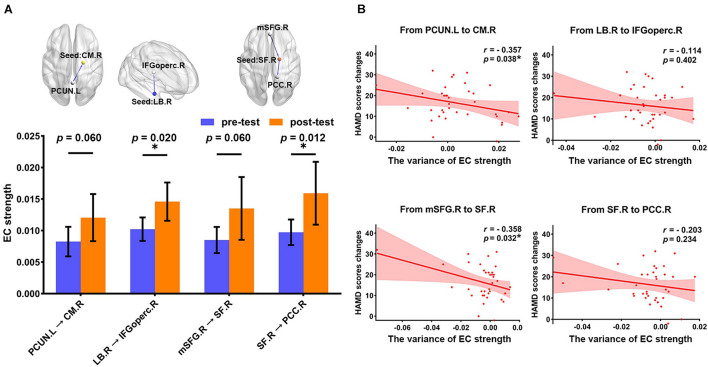
The association of the changes in two different assessment methods between pre- and post-test of pharmacotherapy. **(A)** The histogram shows the variance in the EC strength between pre- and post-test of pharmacotherapy (FDR correction at *p* < 0.05), including the EC from the left PCUN to the right CM (*t* = 1.942, *p* = 0.060), the EC from the right LB to the right IFGoperc (*t* = 2.444, *p* = 0.020), the EC from the right mSFG to the right SF (*t* = 1.941, *p* = 0.060), and the EC from the right SF to the right PCC (*t* = 2.639, *p* = 0.012). *p < 0.05. **(B)** Scatter plots for the relationship between HAMD scores changes and the variance of EC strength. The significant correlation was found in the EC from the left PCUN to the right CM (*r* = –0.357, *p* = 0.038) and the EC from the right mSFG to the right SF (*r* = –0.358, *p* = 0.032). *p < 0.05. EC, effective connectivity; PCUN, precuneus; CM, centromedial amygdala; LB, basolateral amygdala; IFGoperc, opercular part of inferior frontal gyrus; mSFG, medial superior frontal gyrus; SF, superficial amygdala; PCC, posterior cingulate cortex; HAMD, 24-item Hamilton Rating Scale for Depression; L, left hemisphere; R, right hemisphere.

The longitudinal results showed the variance of EC strength between pre- and post-test of pharmacotherapy. As shown in Figure 3A, through paired-sample *t*-test, we have found that all abnormal EC of right amygdala subregions had a normalizing effect after treatment. The EC from the right LB to the right IFGoperc (*t* = 2.444, *p* = 0.020) and the EC from the right SF to the right PCC (*t* = 2.639, *p* = 0.012) showed significant differences in pretest and post-test. The variance of EC strength in the remaining connectivity approached significance, which is the EC from the left PCUN to the right CM (*t* = 1.942, *p* = 0.060) and from the right mSFG to the right SF (*t* = 1.941, *p* = 0.060), respectively.

Correlation analysis between the variance of mean EC strength and HAMD score changes is illustrated in Figure 3B. We found that improvement in symptoms after 12-week treatment was significantly correlated with the variance of mean EC strength in the EC from the left PCUN to the right CM (*r* = −0.357, *p* = 0.038) and the EC from the right mSFG to the right SF (*r* = −0.358, *p* = 0.032). There was no significant correlation in the EC from the right LB to the right IFGoperc (*r* = −0.144, *p* = 0.402) and the EC from the right SF to the right PCC (*r* = −0.203, *p* = 0.234), but the change direction of them is still consistent with the change direction of symptom improvement.

### Predictive Accuracy

**FIGURE 4:**
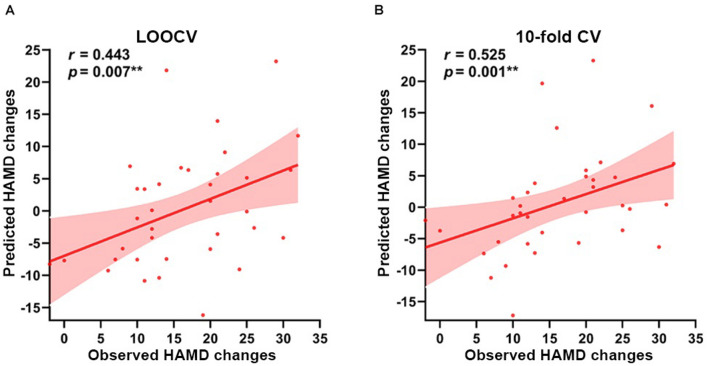
Prediction results based on the EC of right amygdala subregions at baseline for symptom improvement. Scatter plots show the Spearman’s rank correlations between the observed HAMD scores changes and predicted HAMD scores changes of all participants in **(A)** LOOCV: *r* = 0.443, permutation *p* = 0.007, and **(B)** 10-fold CV: *r* = 0.525, permutation *p* = 0.001, respectively. **p < 0.01. LOOCV, Leave-one-out cross-validation; 10-fold CV, 10-fold cross-validation; HAMD, 24-item Hamilton Rating Scale for Depression.

Based on the EC of right amygdala subregions at baseline as features, the predicted model yielded significant prediction power. As Figure 4 shows, the model analysis revealed that the EC in right amygdala subregions exhibited excellent performance in predicting symptom improvement in MDD patients; the predicted HAMD changes had a Spearman’s rank correlation of *r* = 0.443 with the observed HAMD changes with a nested LOOCV (permutation *p* = 0.007), and Spearman’s rank correlation is *r* = 0.525 in a nested 10-fold CV (permutation *p* = 0.001).

## Discussion

In the current study, we investigated the abnormal EC patterns of right amygdala subregions in MDD based on GCA. Three abnormal EC pathways of right amygdala subregions were identified in baseline MDD patients. With the relief of symptoms, these abnormal connections are also normalized following 12-week pharmacological therapy, and correlation analysis results revealed that the variance of EC strength is related to the change of HAMD scores. More importantly, individual depressive symptom improvement can be predicted using the EC of right amygdala subregions at baseline.

### Abnormal Effective Connectivity

The GCA provided a new tool for exploring the EC, and emerging reports have suggested that GCA can effectively identify the directed functional interactions between brain regions (Chen et al., 2009). We found the abnormal EC of depressed individuals mainly in three significant inhibitory pathways, that is, from the left PCUN to the right CM, from the right LB to the right opercular part of IFG, and from the right mSFG to the right SF to the right PCC. These abnormal brain regions provided preliminary evidence for the neural substrate of clinical symptom, which promotes further analysis.

Previous evidence suggested that the mSFG plays an important role in emotional processing and response and has been implicated in some emotional disorders (Phan et al., 2003). For example, our previous research found that the FC between mSFG and medial orbitofrontal cortex (mOFC) subregions is correlated with anxiety in healthy male adults (Xue et al., 2018). The activity of the mSFG is related to the amygdala, which suggested that the extent of coupling between the mSFG and the amygdala associated with emotional response to negative scenes (Wu et al., 2016). The SF of the amygdala is also considered to be involved in social and affective information processing (Adolphs, 2008). The finding of inhibitory connection from the mSFG to the SF supported the existing theories suggesting that the neural mechanism of depression has extensive involvement within frontolimbic circuitry (Lai, 2019). Besides, our results also found that the function of PCC is inhibited by the right SF, and the right CM is inhibited by the PCUN in MDD patients. The PCC and the adjacent PCUN are the main components of the default mode network (DMN), a network of brain regions that are more active when the brain is at rest. A large number of neuroimaging studies pointed out that the DMN is closely associated with neuropathological mechanism of MDD (Kaiser et al., 2015). Consistent with our results, the abnormal connectivity between the amygdala and the PCC or the PCUN has been found in different MDD populations (Cullen et al., 2016). Furthermore, similar to the function of the right amygdala, the PCC and the PCUN also have the advantages in visual imaging processing (Shen et al., 2015). Due to the fact that the right CM mediates behavioral responses to potentially harmful stimuli, taken together, one interpretation of our findings is that the damage of frontolimbic circuitry may inhibit the normal function of the DMN in emotional processing for external stimulus, and then inhibiting the CM nucleus of the right amygdala makes correct emotional responses and cannot habituate from a negative emotional stimulus up to wrong emotional responses, such as sustaining negative emotions and thinking rumination. Additionally, the IFG is involved in relaying top-down cognitive inputs, which was shown to be involved in the updating of task representations and to be activated commonly in multiple cognitive tasks (Derrfuss et al., 2005; Hampshire et al., 2010). The IFG has connected with the limbic system and played a key role in cognitive-emotional integration and continuous behavioral monitoring (Gao et al., 2016). We speculated that the LB nucleus of the right amygdala, as the input region, may be due to inadequate high-level cognitive guidance, resulting in lower-level emotional cognitive processing disorders.

### Longitudinal EC Analyses Following 12-Week Pharmacotherapy

The normalization of aberrant right amygdala subregions connectivity may indicate that the EC of right amygdala subregions correlated with the depressive symptoms. The longitudinal analyses following 12-week pharmacotherapy revealed that the depressive symptoms and hypoconnectivities of right amygdala subregions were ameliorated with pharmacotherapy; in other words, the pharmacotherapy may relief the symptom of depression by improving the right amygdala subregion brain function. This claim echoed previous studies, which revealed that the pharmacological treatment improved the SF nucleus function for emotion and social information processing and the ability of the CM nucleus to make correct emotion responses (Phan et al., 2003). Another study suggested that pharmacological antidepressant effects can be measured in terms of some increase of frontolimbic connectivity and that these effects were most clearly demonstrated by the change of amygdala connections (Chen et al., 2008). Considering that mSFG and IFGoperc are part of frontolimbic circuitry and are critical for the pathological mechanism of MDD, SSRIs therapy may, in part, improve depressive symptoms by restoring the connectivity between the amygdala and these regions. Some analyses exploring the improvement of symptom predictors for pharmacotherapy have found the potential of the PCC as predictive of improved response (Rizvi et al., 2013). Lower baseline EC between the CM and the PCC exhibited increased strength after treatment in our study, which implies that this connectivity was associated with treatment response to SSRIs.

Correlation analysis further supports our conclusion, which shows that depressive symptom improvement is associated with the change of brain function or neuroplasticity. The relationship between MDD improvement and the PCUN component of the DMN has been identified, which is consistent with previous reports of the DMN being associated with symptom improvement (Fonseka et al., 2018). The PCUN and the PCC located in the midline cortical regions of the DMN mediate self-referential processing; they may have excessive involvement in information processing in MDD (Li et al., 2013). What is more, SSRI selective altered intrinsic regional DMN connectivity has also been found in previous research (van de Ven et al., 2013). The result of paired-sample *t*-test between before and after treatment shows that the EC of the IFGoperc and the PCC with right amygdala subregions was reconfigured under the influence of treatment, although no significant corrections were found there, which is related to the mechanism of pharmacotherapy. MDD is a complex mental disease, which involves a variety of abnormal brain functions (Furey et al., 2013). The HAMD score only reflects the part of clinical symptoms in MDD patients, and there are still many potential abnormalities in MDD that may be changed by treatment. In addition, due to the internal heterogeneity of mental illness itself and the influence of external environment, the clinical diagnosis is often not as detailed as research projects (Gao et al., 2018). Some hidden anomalies that are not related to the HAMD scores in MDD could be found by objective brain imaging measurements. This is because the function of the human brain, which is characterized by complex spatial structure, may have some complex non-linear relationship with clinical scores that cannot be found in correlation analysis (Cohen et al., 2020). Therefore, their changes are still meaningful for better understanding of the neural mechanism of MDD treatment. These results indicated that right amygdala subregion function was suggested to be relevant to characterize the neurobiology of antidepressant medications and may be useful in guiding treatment selection in future studies.

### Predictive Power of Effective Connectivity

There is no doubt that the etiopathogenesis of depression has its biological basis (Ge et al., 2020). It is important to identify the neurobiological mechanisms of pharmacotherapy in MDD, so that this knowledge can be applied to improve clinical treatment. Great progress has been made in the study of human brain dysfunction caused by depression or MDD using MRI (Dosenbach et al., 2010). However, whether these human brain functional indicators contain enough information to help us predict the therapeutic improvement remains a big unknown. Along this line, many studies try to explore useful therapeutic predictors using difference MRI neuroimaging makers and machine-learning algorithms (Jiang et al., 2018). For example, a previous study indicated that the right amygdala was associated with MDD, and the right amygdala connectivity predicted the psychotherapy improvement in depressed adolescents (Straub et al., 2017). In our study, our results also demonstrated the predictive ability of EC of right amygdala subregions at baseline for symptom improvement after 12-week pharmacological therapy in MDD. Consistent with our hypothesis, these findings are suggestive of the fact that right amygdala subregion-seeded EC may aid in understanding mechanisms of pharmacotherapy in MDD and holds the promise for future research to improve the clinical outcomes. Since emotional dysfunctions are the main symptom of MDD, it makes sense to predict the symptom improvement from the amygdala at baseline, and many studies have discovered the potential of the amygdala as a predictor for pharmacotherapy (Furey et al., 2013; Williams et al., 2015). Moreover, the correlation between roles of the right amygdala for visual emotional stimuli and MDD symptoms improvement is supported by some studies (Furey et al., 2013; Szczepanik et al., 2016).

Employing internal validation procedures, we built our predictive models based on two different cross-validation methods. Encouragingly, the results are robust and still support our conclusion. From the methodological principle of GCA, the “scrubbing” in data preprocessing removes the “bad” time points and may affect the evaluation results of GCA. We thus repeated all analyses without “scrubbing” in the preprocessing analysis. Our findings were similar, and the model maintains good prediction performance. The high reproducibility shows the feasibility of the EC in the right amygdala subregions for predicting the symptom improvement of MDD. Predictive data mining has become very popular in neuroimaging research, especially in the study of mental diseases (Meng et al., 2017). The ultimate purpose of neuroimaging diagnosis is to predict the symptom of patients, and our results strengthen the role of the amygdala in the pathophysiology of MDD and its importance in model mood dysregulation and as a new therapeutic target.

### Limitation

Several limitations warrant further consideration. First, the number of participants was small, and not all subjects have completed the pre- and post-test, which may limit the statistical power in finding the abnormal EC brain regions and challenge our results. Therefore, a larger number of sample sizes and measures to prevent the loss of subjects are needed in further studies. Second, there is internal heterogeneity of SSRIs in the pharmacological drugs among patients for the current dataset. However, only the baseline data of R-fMRI have been used to predict the symptom improvement, and these data are not related to heterogeneous pharmacotherapy. Consistent with our dataset and previous studies on the prediction of the MDD symptom improvement treated with heterogenous antidepressant drugs, neuroimaging markers also perform effective predictive capacity (Shen et al., 2015), and a large-sample study shows that there are only subtle differences between different pharmacological treatment modalities (Stassen et al., 2007). Nevertheless, to control the heterogeneity of therapy medicine is needed for future research. Third, the dosages of drugs could affect the experimental results. As the longitudinal study leads to the loss of participant data, only 15 of the 36 participants in our remaining data have complete information about the dosages of drugs. We repeated our analysis by using the EC in right amygdala subregions of these 15 subjects as features and incorporate the drug dose as a covariant in the predicted model. Fortunately, the EC of right amygdala subregions still significant predict the symptom improvement. The high reproducibility of our findings indicated the reliability of right amygdala subregions-based predictor for treatment outcomes, but a more complete large sample size should be used to verify this conclusion in future research. Finally, the current study revealed that the EC in right amygdala subregions significantly predicts the symptom improvement of MDD patients after 12-week pharmacological therapy. However, apart from the treatment improvement after 12-week pharmacotherapy, an important topic in the future is whether the symptom improvement of other treatment courses can be predicted.

## Conclusion

The current study demonstrated the abnormal right amygdala subregion-seeded EC, and the results mainly concentrated in the frontolimbic circuits and the DMN. The longitudinal analysis found that the symptom improvement caused by antidepressant medications is associated with the change of mean EC strength of right amygdala subregions in MDD patients. Importantly, the EC in right amygdala subregions at baseline significantly predicts the symptom improvement of pharmacotherapy. The function of right amygdala subregions may contribute to a better understanding of the neurobiological mechanism of pharmacotherapy. Meanwhile, these results also provided new supporting evidence for the application of neuroimaging techniques in the treatment outcome prediction and thus guide more individualized treatment for MDD patients.

## Data Availability Statement

The raw data supporting the conclusions of this article will be made available by the authors, without undue reservation.

## Ethics Statement

The studies involving human participants were reviewed and approved by the Institutional Review Boards of Hangzhou Normal University. The patients/participants provided their written informed consent to participate in this study.

## Author Contributions

YX, DW, and S-WX had full access to all of the data in the study and take responsibility. S-WX, YX, DW, and ZT proposed the study concept and designed the experiments. YX, S-WX, and LZ drafted the manuscript. YX, S-WX, LZ, and ZL modified the manuscript for important intellectual content. YX, CK, HL, and CP were responsible for statistical analysis. DW and S-WX obtained funding. YX, XH, SF, and YW were responsible for administrative, technical, or material support. All authors participated in acquisition, analysis, or interpretation of data and contributed to the article and approved the final manuscript.

## Conflict of Interest

The authors declare that the research was conducted in the absence of any commercial or financial relationships that could be construed as a potential conflict of interest.

## Publisher’s Note

All claims expressed in this article are solely those of the authors and do not necessarily represent those of their affiliated organizations, or those of the publisher, the editors and the reviewers. Any product that may be evaluated in this article, or claim that may be made by its manufacturer, is not guaranteed or endorsed by the publisher.

## Footnotes

1
http://www.rfmri.org/
